# Supplementary material for: High-level cognition during story listening is reflected in high-order dynamic correlations in neural activity patterns
Source: Nat Commun. 2021 Sep 30;12:5728. doi: 10.1038/s41467-021-25876-x (PMC8484677; doi:10.1038/s41467-021-25876-x)
Supplement: Supplementary file 1 — Supplementary Information [file 41467_2021_25876_MOESM1_ESM.pdf]

Supplementary materials for: High-level cognition during story listening is reflected in high-order dynamic correlations in neural activity patterns

Lucy L. W. Owen[1], Thomas H. Chang[1,2], and Jeremy R. Manning[1,†]

[1]Department of Psychological and Brain Sciences,
Dartmouth College, Hanover, NH
[2]Amazon.com, Seattle, WA
[†]Address correspondence to jeremy.r.manning@dartmouth.edu

August 5, 2021

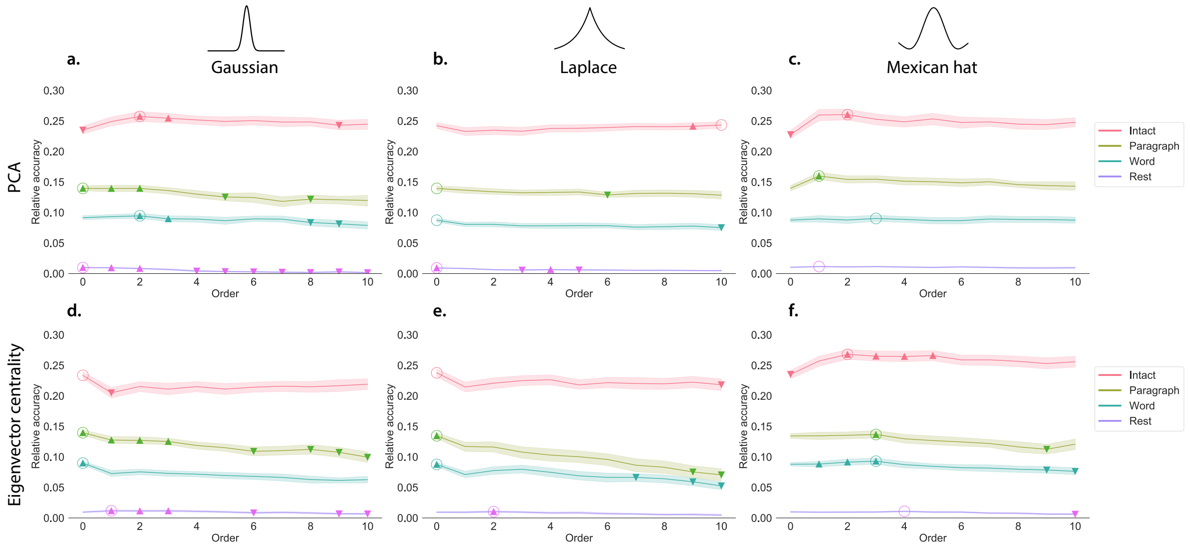

Figure S1: Across-participant timepoint decoding accuracy varies with correlation order and cognitive engagement across kernels. a.-c. Timepoint decoding accuracy as a function of order: PCA. *Order* (x-axis) refers to the maximum order of dynamic correlations that were available to the classifiers (see *Feature weighting and testing*). The reported across-participant decoding accuracies for **a. Gaussian**, **b. Laplace**, and **c. Mexican hat** kernel shapes are averaged over all widths (see *Identifying robust decoding results*). The y-values are displayed relative to chance accuracy (intact: $\frac{1}{300}$; paragraph: $\frac{1}{272}$; word: $\frac{1}{300}$; rest: $\frac{1}{400}$). The error ribbons denote 95% confidence intervals of the mean across cross-validation folds (i.e., random assignments of participants to the training and test sets). The colors denote the experimental condition. Arrows denote sets of features that yielded reliably higher (upward facing) or lower (downward facing) decoding accuracy than the mean of all other features (via a two-tailed t-test, thresholded at $p < 0.05$; exact values are reported in the Source Data file). The circled values represent the maximum decoding accuracy within each experimental condition. Panels a.-c. used PCA to project each high-dimensional pattern of dynamic correlations onto a lower-dimensional space. **d.-f. Timepoint decoding accuracy as a function of order: eigenvector centrality.** These panels are in the same format as Panel a.-c., but here eigenvector centrality has been used to project the high-dimensional patterns of dynamic correlations into lower-dimensional spaces. Source data are provided as a Source Data file.

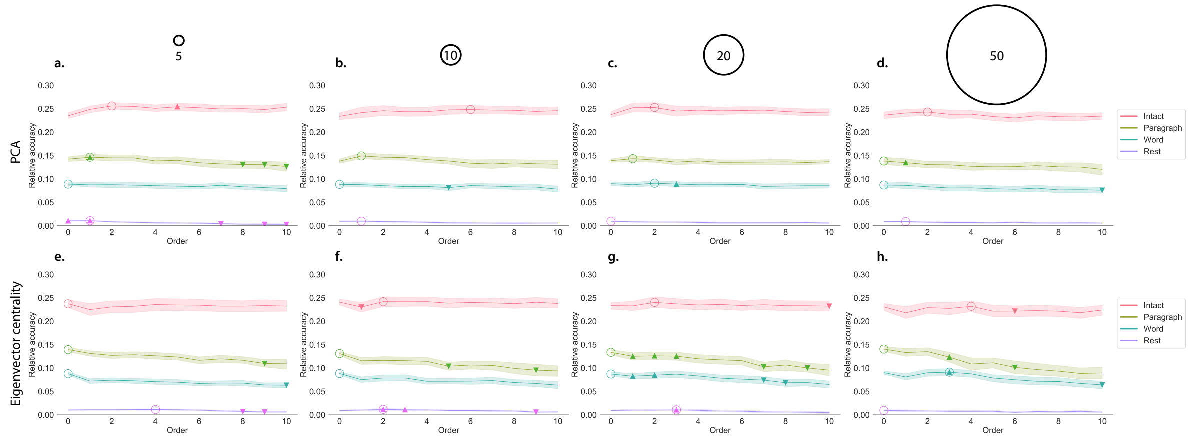

Figure S2: Across-participant timepoint decoding accuracy varies with correlation order and cognitive engagement across widths. a.-d. Timepoint decoding accuracy as a function of order: PCA. *Order* (x-axis) refers to the maximum order of dynamic correlations that were available to the classifiers (see *Feature weighting and testing*). The reported across-participant decoding accuracies for kernel widths of **a. 5**, **b. 10**, **c. 20**, and **d. 50** are averaged over all kernel shapes (see *Identifying robust decoding results*). The y-values are displayed relative to chance accuracy (intact: $\frac{1}{300}$; paragraph: $\frac{1}{272}$; word: $\frac{1}{300}$; rest: $\frac{1}{400}$). The error ribbons denote 95% confidence intervals of the mean across cross-validation folds (i.e., random assignments of participants to the training and test sets). The colors denote the experimental condition. Arrows denote sets of features that yielded reliably higher (upward facing) or lower (downward facing) decoding accuracy than the mean of all other features (via a two-tailed t-test, thresholded at $p < 0.05$; exact values are reported in the Source Data file). The circled values represent the maximum decoding accuracy within each experimental condition. Panels a.-d. used PCA to project each high-dimensional pattern of dynamic correlations onto a lower-dimensional space.**e.-h. Timepoint decoding accuracy as a function of order: eigenvector centrality.** These panels are in the same format as Panel a.-d., but here eigenvector centrality has been used to project the high-dimensional patterns of dynamic correlations into lower-dimensional spaces. Source data are provided as a Source Data file.

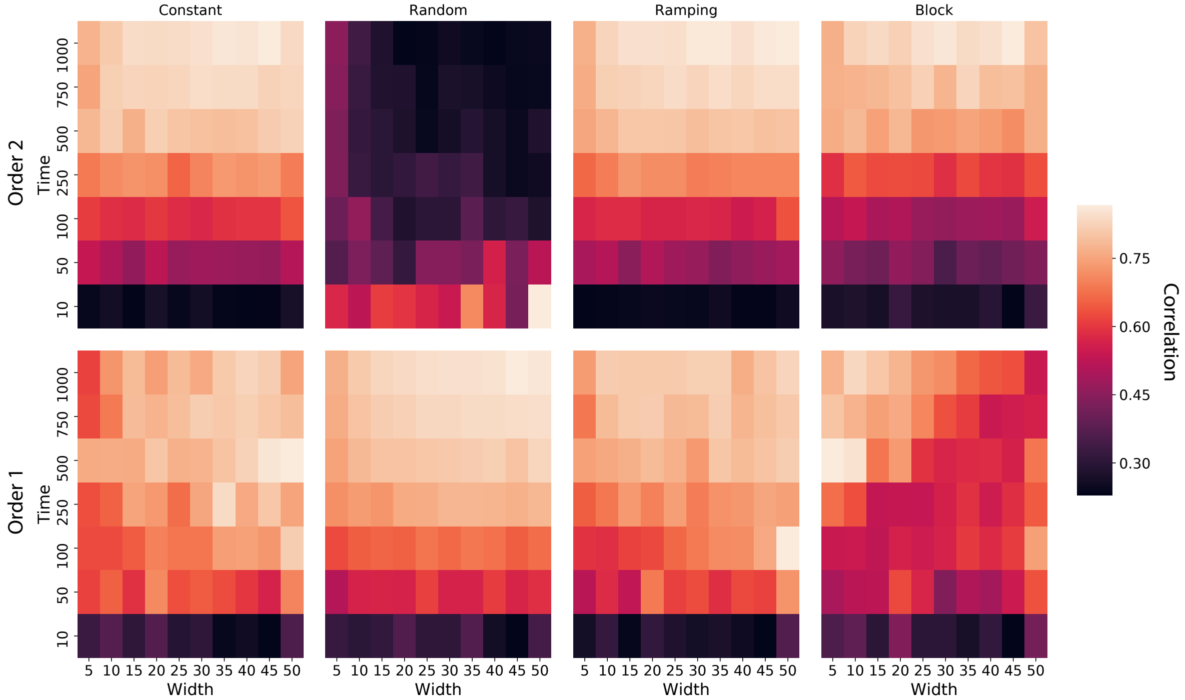

Figure S3: Recovery heatmap of simulated first-order and second-order dynamic correlations across time and widths. Each panel displays a heatmap of the average correlations between the vectorized upper triangles of the recovered first-order and second-order correlation matrices and the true (simulated) first-order and second order correlation matrices. The averages are taken across several randomly generated synthetic datasets for each timeseries pattern (constant, random, ramping, and data; see *Synthetic data: simulating dynamic higher-order correlations*). The x-axes of each heatmap denote varying Laplace-shaped kernel widths (see Figs. S1 and S2), and the y-axes of each heatmap denote varying durations (in samples) of the synthetic datasets. A total of 10 synthetic datasets were generated for each duration (row) and timeseries pattern (panel). Source data are provided as a Source Data file.

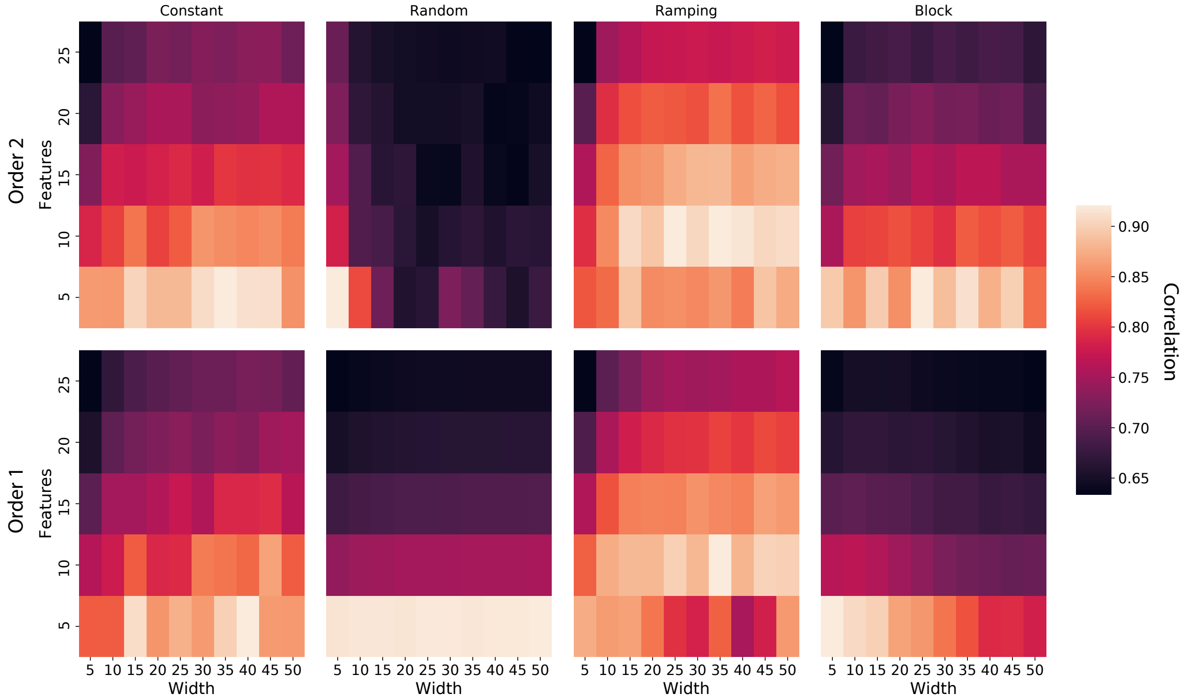

Figure S4: Recovery heatmap of simulated first-order and second-order dynamic correlations across features and widths. Each panel displays a heatmap of the average correlations between the vectorized upper triangles of the recovered first-order and second-order correlation matrices and the true (simulated) first-order and second order correlation matrices. The averages are taken across several randomly generated synthetic datasets for each timeseries pattern (constant, random, ramping, and data; see *Synthetic data: simulating dynamic higher-order correlations*). The x-axes of each heatmap denote varying Laplace-shaped kernel widths (see Figs. S1 and S2), and the y-axes of each heatmap denote varying numbers of features (K) used in each of the synthetic datasets. A total of 10 synthetic datasets were generated for each number of features (row) and timeseries pattern (panel). Source data are provided as a Source Data file.

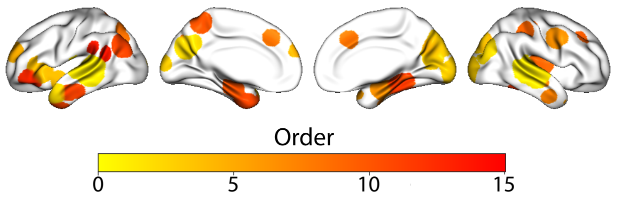

Order 1	Order 2	Order 3	Order 4	Order 5
voice	voice	cuneus	anterior insula	integrate
speech	precuneus	v1	insula inferior	spatially
superior temporal	temporal	visual	inferior frontal	anterior hippocampus
listening	speaker	early visual	insula	occipital
temporal gyrus	temporal gyrus	primary visual	executive	recollection
sts	spoken	lingual gyrus	inferior	orbital
speech perception	heard	occiplital	frontal	hippocampal
spoken	sounds	lingual	pole	visual stream
superior	comprehension	visual cortex	frontal gyrus	visual cortex
temporal sulcus	speech perception	integrate	asd	visual

Order 6	Order 7	Order 8	Order 9	Order 10
putamen	hippocampal	face recognition	monitoring	posterior insula
face ffa	hippocampus	recognition	error	insular
ffa	amygdala hippocampus	face	conflict	extrastriate
subsequent	anterior hippocampus	fusiform face	task	insular cortex
fusiform face	medial temporal	face ffa	frontal cortex	lateral occipital
images	parahippocampal	ffa	read	letter
selective	mtl	faces	errors	occipital
basal ganglia	temporal lobe	fusiform gyrus	insula anterior	visual
ganglia	parahippocampal cortex	hippocampal	color	painful
basal	lobe	medial temporal	demands	insula

Order 11	Order 12	Order 13	Order 14	Order 15
memory encoding	angular gyrus	parahippocampal	inferior frontal	motor task
subsequent	angular	navigation	inferior	sensorimotor cortex
hippocampus	anterior temporal	objects	words	contralateral
hippocampal	temporal lobes	parahippocampal cortex	pseudowords	hand
associative	default	parahippocampal gyrus	frontal gyrus	sensorimotor
memory	default mode	anterior hippocampus	semantic	finger
lobe mtl	network dmn	encoding	reading	primary
mtl	self referential	memory encoding	chinese	spatially
retrieval	dmn	fusiform	word	tapping
episodic	default network	face ffa	orthographic	index finger

Figure S5: Top terms associated with the most strongly correlated nodes at each order, for the *intact* experimental condition. Each color corresponds to one order of inter-subject functional correlations. The inflated brain plots display the locations of the endpoints of the 10 strongest (absolute value) correlations at each order, projected onto the cortical surface[S1]. The lists of terms display the top 10 Neurosynth terms[S2] decoded from the corresponding brain maps for each order. (Also see Fig. 6, top row, in the main text.) Source data are provided as a Source Data file.

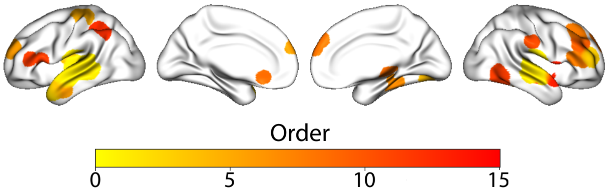

Order 1	Order 2	Order 3	Order 4	Order 5
sounds	voice	auditory cortex	gyri	occipital
auditory cortex	precuneus	sound	temporo	extrastriate
listening	temporal	sounds	posterior temporal	visual
auditory	speaker	auditory	posterior middle	reaching
listened	temporal gyrus	heschl gyrus	temporo parietal	objects
auditory stimuli	spoken	heschl	visual stream	arm
speech	heard	acoustic	secondary somatosensory	dlpfc
primary auditory	sounds	gyrus stg	insular	incorrect
sound	comprehension	planum	verb	occipital gyrus
acoustic	speech perception	listened	temporal gyri	index finger

Order 6	Order 7	Order 8	Order 9	Order 10
anterior temporal	fusiform	ventrolateral	chinese	prefrontal
mental states	object	fusiform gyrus	reading	prefrontal cortex
lateral temporal	memory encoding	inferior frontal	naming	dorsolateral prefrontal
tom	fusiform gyrus	inferior	judgment	cortex precuneus
pole	fusiform gyrus	fusiform	letter	precuneus posterior
temporal pole	venral visual	intentions	english	medial
comprehension	faces	pfc	speakers	dorsolateral
virtual	reaching	cognitive impairment	outcomes	precuneus
mind tom	parahippocampal gyrus	semantic	ventral striatum	dorsomedial prefrontal
theory of mind	objects	mild cognitive	nucleus accumbens	medial prefrontal

Order 11	Order 12	Order 13	Order 14	Order 15
tactile	ifg	inferior parietal	musical	tom
secondary somatosensory	gyrus ifg	parietal lobule	anterior superior	mind tom
precuneus	viewing	working memory	superior temporal	temporal
somatosensory cortex	angular gyrus	ipl	listening	medial
pain	v5	working	spoken	temporal sulcus
temporo parietal	angular	anterior intraparietal	music	dorsomedial prefrontal
primary somatosensory	motion	parietal cortex	comprehension	theory mind
somatosensory	social interaction	frontoparietal	voice	sts
somatosensory cortices	visual	fronto parietal	speech	dorsomedial
precuneus posterior	temporo parietal	posterior parietal	frontal operculum	medial prefrontal

Figure S6: Top terms associated with the most strongly correlated nodes at each order, for the *paragraph* experimental condition. This figure is in the same format as Figure S5, but displays results for the paragraph-scrambled story listening condition. (Also see Fig. 6, second row, in the main text.) Source data are provided as a Source Data file.

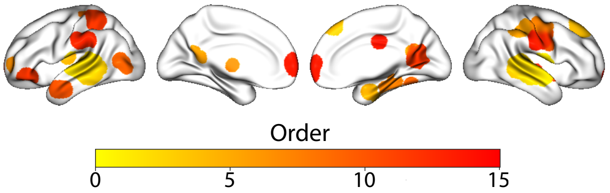

Order 1	Order 2	Order 3	Order 4	Order 5
listening	voice	temporal gyrus	anterior temporal	mild cognitive
speech	temporal gyrus	voice	expression	memory
voive	sounds	superior temporal	lateral temporal	medial temporal
sounds	listening	temporal sulcus	temporal lobes	lobe
speeck perception	speech	speaker	lobes	mtl
spoiken	spoken	sts	temporal lobe	retrieval
listened	audisotry	speech	fearful faces	episodic
audiory	superior temporal	listening	temporal	temporal lobe
superior temporal	listened	spoken	lobe	lobe mtl
temporal gyrus	speech perception	superior	neutral faces	episodic memory

Order 6	Order 7	Order 8	Order 9	Order 10
inferior parietal	anterior temporal	parahippocampal	face	extrastriate
default mode	amygdala insula	episodic	fusiform face	occipitotemporal cortex
default	expression	parahippocampal gyrus	face ffa	occipitotemporal
inhibition	amygdala	hippocampus	ffa	videos
pcc	anterior prefrontal	hippocampal	fusiform	v5
posterior cingulate	neutral faces	memory	faces	visual
response inhibition	temporal lobbe	medial temporal	fusiform gyrus	mt
medial	anxiety	anterior hippocampus	reaching	perception
autobiographical	fearful faces	episodic memory	occipital	fusiform
dmn	amygdala responses	temporal lobe	face recognition	objects

Order 11	Order 12	Order 13	Order 14	Order 15
primary somatosensory	medial prefrontal	reaching	locations	medial
premotor	medial	retrosplenial	precuneus	posterior cingulate
anterior temporal	anterior temporal	visuomotor	body	pcc
somatosensory	prefrontal cortex	tools	supramarginal	medial prefrontal
premotor cortex	prefrontal	precuneus	videos	cortex precuneus
inferior parietal	semantic memory	videos	actions	referential
s1	negative positive	touch	mt	self referential
ventral premotor	mpfc	cortex precuneus	video clips	cortex posterior
execution	autobiographical	navigation	video	autobiographical
somatosensory cortex	temporal lobe	retrosplenial cortex	occipitotemporal	hippocampus

Figure S7: Top terms associated with the most strongly correlated nodes at each order, for the *word* experimental condition. This figure is in the same format as Figure S5, but displays results for the word-scrambled story listening condition. (Also see Fig. 6, third row, in the main text.) Source data are provided as a Source Data file.

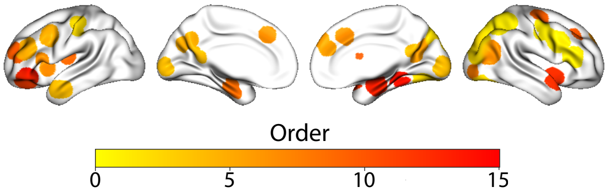

Order 1	Order 2	Order 3	Order 4	Order 5
cortex parietal	occipital	parietal	default	dorsolateral
intraparietal	visual	conflict	autobiographical	frontal
parietal cortex	etrastriate	task	default mode	middle frontal
ips	vision	visual cortex	posterior cingulate	motion
posterior parietal	occipital cortex	working memory	anterior temporal	mt
parietal	visual cortex	supplementary motor	dmn	verbal
intraparietal sulcus	visual stream	working	early visual	memory
symbbolic	motion	arithmetic	mental imagery	visual motion
superior parietal	v1	supplementary	primary visual	dorsolateral prefrontal
prefrontal parietal	mt	anterior temporal	autobiographical memory	vision

Order 6	Order 7	Order 8	Order 9	Order 10
locations	insual anterior	occipital	posterior insula	inferior occipital
navigation	error	precuneus	temporal lobe	anterior prefrontal
medial	anterior insula	letter	mtl	face
medial prefrontal	monitoring	inferior occipital	amygdala responses	selective
orienting	insula	visual	lobe mtl	face ffa
precuneus	conflict	cortex precuneus	insula	dlpfc
memory retrieval	frontal cortex	occipital cortex	lobe	ffa
location	insular	lateral occipital	mild cognitive	fusiform face
retrieval	taste	spatially	insular	dorsolateral
dorsomedial	errors	sulcus	cognitive impairment	cortex dlpfc

Order 11	Order 12	Order 13	Order 14	Order 15
hippocampal	food	insula	amygdala hippocampus	s1
hippocampus	ofc	taste	hippocampal	striatal
anterior hippocampus	cortex ofc	insular	hippocampus	outcomes
lobe	orbitofrontal cortex	insular cortex	anterior hippocampus	monetary
amygdala hippocampus	nucleus accumbens	unpleasant	medial temporal	memory encoding
parahippocampal gyrus	accumbes	pleasant	amygdala	accumbens
mtl	orbitofrontal	amygdala	parahippocampal	mesolimbic
medial temporal	value	amygdala insula	navigation	nucleus accumbens
temporal lobe	orbital	posterior insula	unpleasant	frontal
lobe mtl	hypothalamus	pain	episodic	money

Figure S8: Top terms associated with the most strongly correlated nodes at each order, for the *rest* experimental condition. This figure is in the same format as Figure S5, but displays results for the resting state condition. (Also see Fig. 6, bottom row, in the main text.) Source data are provided as a Source Data file.

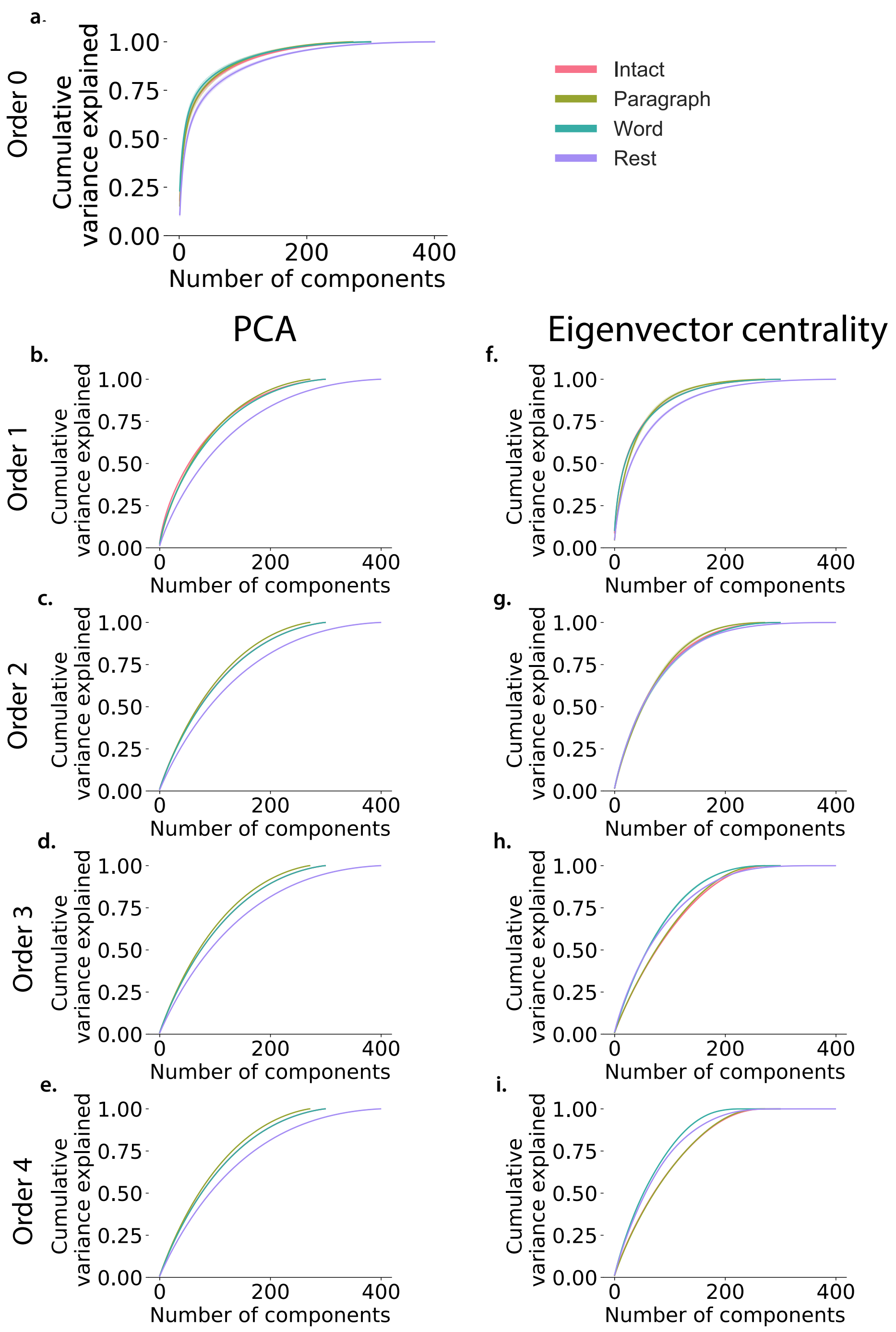

Figure S9: Cumulative percent variance explained as a function of the number of principle components. *Order* refers to the order of the dynamic correlations calculated. Principle components analysis was performed, and reduced independently for each subject. Maximum number of components varies with the total time for each condition (intact: 300; paragraph: 272; word: 300; rest: 400). **a. Cumulative percent variance as a function of number of components for Order 0.** PCA was performed on the raw activity patterns (Order 0). **b.-e. Cumlative percent variance as a function of number of components for Orders 1.-4.: PCA** Dynamic correlation were calculated for orders 1-4 using PCA to project each high-dimensional pattern of dynamic correlations onto a lower-dimensional space. **f.-i. Cumlative percent variance as a function of number of components for Orders 1.-4.: eigenvector centrality.** These panels are in the same format as Panel b.-e., but here eigenvector centrality has been used to project the high-dimensional patterns of dynamic correlations onto a lower-dimensional space. Source data are provided as a Source Data file.

Supplementary references

[S1] Combrisson, E. *et al.* Visbrain: a multi-purpose GPU-accelerated open-source suite for multimodal brain data visualization. *Frontiers in Neuroinformatics* **13**, 1–14 (2019).

[S2] Rubin, T. N. *et al.* Decoding brain activity using a large-scale probabilistic functional-anatomical atlas of human cognition. *PLoS Computational Biology* **13**, e1005649 (2017).
