# Supplementary material for: High-level cognition during story listening is reflected in high-order dynamic correlations in neural activity patterns
Source: Nat Commun. 2021 Sep 30;12:5728. doi: 10.1038/s41467-021-25876-x (PMC8484677; doi:10.1038/s41467-021-25876-x)
Supplement: Supplementary file 3 — Reporting Summary [file 41467_2021_25876_MOESM3_ESM.pdf]

nature research

Corresponding author(s): Jeremy R. Manning

Last updated by author(s): Aug 5, 2021

Reporting Summary

Nature Research wishes to improve the reproducibility of the work that we publish. This form provides structure for consistency and transparency in reporting. For further information on Nature Research policies, see Authors & Referees and the Editorial Policy Checklist .

Statistics

For all statistical analyses, confirm that the following items are present in the figure legend, table legend, main text, or Methods section.

n/a	Confirmed	
☐	☒	The exact sample size (*n*) for each experimental group/condition, given as a discrete number and unit of measurement
☐	☒	A statement on whether measurements were taken from distinct samples or whether the same sample was measured repeatedly
☐	☒	The statistical test(s) used AND whether they are one- or two-sided<br>*Only common tests should be described solely by name; describe more complex techniques in the Methods section.*
☐	☒	A description of all covariates tested
☐	☒	A description of any assumptions or corrections, such as tests of normality and adjustment for multiple comparisons
☐	☒	A full description of the statistical parameters including central tendency (e.g. means) or other basic estimates (e.g. regression coefficient) AND variation (e.g. standard deviation) or associated estimates of uncertainty (e.g. confidence intervals)
☐	☒	For null hypothesis testing, the test statistic (e.g. *F*, *t*, *r*) with confidence intervals, effect sizes, degrees of freedom and *P* value noted<br>*Give P values as exact values whenever suitable.*
☒	☐	For Bayesian analysis, information on the choice of priors and Markov chain Monte Carlo settings
☐	☒	For hierarchical and complex designs, identification of the appropriate level for tests and full reporting of outcomes
☐	☒	Estimates of effect sizes (e.g. Cohen's *d*, Pearson's *r*), indicating how they were calculated

Our web collection on statistics for biologists contains articles on many of the points above.

Software and code

Policy information about availability of computer code

Data collection	We analyzed a public fMRI dataset collected by Simony et al. (2016). It may be downloaded at arks.princeton.edu/ark:/88435/dsp015d86p269k. Functional data were preprocessed and analysed using FSL (www.fmrib.ox.ac.uk/fsl). The preprocessed data are included in the public dataset at the above link.
Data analysis	All algorithms used were described in the paper and coded in Python. Code for carrying out HTFA on fMRI data may be found as part of the BrainIAK toolbox which may be downloaded (https://brainiak.org). We have released an open-source Python toolbox (https://timecorr.readthedocs.io), in tandem with all the code, along with links to the data, needed to replicate the figures in our paper (https://github.com/ContextLab/timecorr-paper). We have included links to these resources in our paper.

For manuscripts utilizing custom algorithms or software that are central to the research but not yet described in published literature, software must be made available to editors/reviewers. We strongly encourage code deposition in a community repository (e.g. GitHub). See the Nature Research guidelines for submitting code & software for further information.

Data

Policy information about availability of data

All manuscripts must include a data availability statement. This statement should provide the following information, where applicable:

- Accession codes, unique identifiers, or web links for publicly available datasets
- A list of figures that have associated raw data
- A description of any restrictions on data availability

We analyzed a public fMRI dataset collected by Simony et al. (2016) that may be downloaded here: arks.princeton.edu/ark:/88435/dsp015d86p269k. We have also published a GitHub repository containing links to download the data, along with code for reading in and analyzing the data in Python: https://github.com/ContextLab/timecorr-paper.

Field-specific reporting

Please select the one below that is the best fit for your research. If you are not sure, read the appropriate sections before making your selection.

☐ Life sciences ☒ Behavioural & social sciences ☐ Ecological, evolutionary & environmental sciences

For a reference copy of the document with all sections, see nature.com/documents/nr-reporting-summary-flat.pdf

Behavioural & social sciences study design

All studies must disclose on these points even when the disclosure is negative.

Study description	We analyzed a public dataset comprising fMRI data (quantitative) collected as participants listened to an auditory recording of a story, listened to a temporally scrambled version of the story, or participated in a resting state scan.
Research sample	The participants in the experiment were members of the Princeton University (and surrounding) community. 36 subjects listened to the intact story (25 females, ages: 18–33). In the paragraph scramble condition there were 17 subjects (6 males, ages: 18–31). In the word scramble condition there were 36 subjects (20 females, ages: 18–33). In the rest condition there were 36 subjects (15 females, ages: 18–30). All subjects were native English speakers with normal hearing and provided written informed consent. Procedures were approved by the Princeton University Committee on Activities Involving Human Subjects, and by the Western Institutional Review Board (Puyallup, WA). All subjects were native English speakers with normal hearing and provided written informed consent.
Sampling strategy	Convenience
Data collection	The primary equipment used to collect the data was a Siemens Skyra MRI scanner. Stimuli were presented using the Psychophysics toolbox (Brainard, 1997; Pelli, 1997). Subjects were provided with an MRI compatible in-ear mono earbuds (Sensimetrics Model S14), which provided the same audio input to each ear. MRI-safe passive noise-canceling headphones were placed over the earbuds, for noise removal and safety. The data were collected by Simony et al. (2016). We report all available information included in the public dataset.
Timing	The dataset was published in 2016, the data generated was from 2015.
Data exclusions	We analyzed all data included in the released dataset.
Non-participation	No participants were excluded or dropped out of the study, as reported in the original paper (Simony et al., 2016)
Randomization	The original study did not report how participants were assigned to experimental conditions.

Reporting for specific materials, systems and methods

We require information from authors about some types of materials, experimental systems and methods used in many studies. Here, indicate whether each material, system or method listed is relevant to your study. If you are not sure if a list item applies to your research, read the appropriate section before selecting a response.

Materials & experimental systems

n/a	Involved in the study	
☒	☐	Antibodies
☒	☐	Eukaryotic cell lines
☒	☐	Palaeontology
☒	☐	Animals and other organisms
☐	☒	Human research participants
☒	☐	Clinical data

Methods

n/a	Involved in the study	
☒	☐	ChIP-seq
☒	☐	Flow cytometry
☐	☒	MRI-based neuroimaging

Human research participants

Policy information about studies involving human research participants

Population characteristics	Intact condition: 36 partiipants (10 female, ages 18--33); paragraph condition: 18 participants (12 female; ages 18--31); word condition: 36 participants (20 female, ages 18--33); rest condition: 36 participants (15 female, ages 18--30).
Recruitment	The original paper did not report how participants were recruited.
Ethics oversight	The experimental protocol was approved by the Princeton University Committee on Activities Involving Human Subjects, and by the Western Institutional Review Board. We report all available information included in the public dataset.

Note that full information on the approval of the study protocol must also be provided in the manuscript.

Magnetic resonance imaging

Experimental design

Design type	The experiment comprised a single stimulus presentation block during which the participant either listened to an auditory recording or lay in the scanner with their eyes open.
Design specifications	Full study details are reported in Simony et al., 2016.
Behavioral performance measures	No behavioral data were analyzed in our study.

Acquisition

Imaging type(s)	Functional
Field strength	3-T
Sequence & imaging parameters	Images were acquired using a T2* weighted echo planer imaging pulse sequence (TR, 1500 ms; echo time, 28 ms; flip angle, 64 degrees), each volume comprising 27 slices of 4 mm thickness; slice-acquisition order was interleaved. In-plane resolution was 3 x 3 mm2 (field of view, 192 x 192 mm2). Anatomical images were acquired using a T1-weighted magnetization-prepared rapid acquisition gradient echo pulse sequence (TR, 2300 ms; echo time, 3.08 ms; flip angle 9!; 0.89 mm3 resolution; field of view, 256 mm2).
Area of acquisition	Whole brain scan

Diffusion MRI ☐ Used ☒ Not used

Preprocessing

Preprocessing software	Functional data were preprocessed and analysed using FSL (www.fmrib.ox.ac.uk/fsl), including correction for head motion and slice-acquisition time, spatial smoothing (6 mm FWHM Gaussian kernel), and high-pass temporal filtering (140 s period).
Normalization	Preprocessed data were aligned to a standard anatomical (MNI152) brain, and interpolated to 3-mm isotropic voxels.
Normalization template	MNI152
Noise and artifact removal	The low-frequency respiratory signal known as 'respiratory variation' (RV) was calculated as the s.d. of the respiratory signal over a sliding window of 3 TRs (9 s), where the center of the window is in the middle of the interval. The experimenters convolved the RV with the respiratory response function in a subject-specific manner, for each of the nine subjects that listened to the intact story and participated in a resting condition. HR can also account for BOLD variance. The experimenters measured the HR by averaging the inter-pulse intervals over 3 TR intervals, converting it to beats per-minute, and convolving it with the cardiac response function. Head motion trajectory. The experimenters calculated the instantaneous head motion for each subject in the breathing group (n=9) as the sum of the absolute displacement (derivative) in the six motion parameters, which were estimated during the motion correction step (translations: Dx, Dy, Dz; rotations:a, b, g). The rotational displacements were converted from degrees to millimeters. Slow changes of respiration over time (RV) have been shown to induce robust changes in the BOLD signal in many areas around the cerebral midline, including parts of the DMN. Therefore, the experimenters used multiple linear regression to project out three nuisance variables from the BOLD data. This was performed separately for each subject and for all conditions before calculating FC or ISFC. Nuisance regressors were: (1) the average time course of high s.d. voxels outside the grey matter mask (voxels in the top 1% largest s.d., likely blood vessels known to be correlated with RV); (2) the average BOLD signal measured in cerebrospinal fluid; (3) the average white matter signal.
Volume censoring	All masks (grey matter, white matter and cerebrospinal fluid) were obtained from the probabilistic FSL atlas in standard MNI space (>95% region probability, Harvard–Oxford cortical and subcortical structural atlases).

Statistical modeling & inference

Model type and settings	We first applied Hierarchical Topographic Factor Analysis (HTFA; Manning et al., 2018; https://brainiak.org/) to the fMRI data. We then applied the model reported in our manuscript to the output of the HTFA model.
Effect(s) tested	We compared neural responses within and across different stimulus presentation blocks as participants listened to an (intact or temporally scrambled) audio story, or underwent a resting state scan.

Specify type of analysis: ☒ Whole brain ☐ ROI-based ☐ Both

Statistic type for inference (See Eklund et al. 2016)	We used an across-participants temporal decoding procedure to compute decoding accuracy given each type (or blend) of neural features.
Correction	We subtracted "chance" decoding accuracy from the initial decoding accuracies we obtained using held-out data.

Models & analysis

n/a	Involved in the study
☐	☒ Functional and/or effective connectivity
☐	☒ Graph analysis
☐	☒ Multivariate modeling or predictive analysis

Functional and/or effective connectivity	We derived a model of dynamic inter-subject functional connectivity (DISFC), reported in the paper. In brief, this method estimates the stimulus-driven dynamic correlations between different regions by comparing data across participants.
Graph analysis	We used principal components analysis and eigenvector centrality to summarize network patterns and compute the relative positions of different nodes in the network. We characterized how these properties changed over time, and whether those changes were similar across participants.
Multivariate modeling and predictive analysis	We used patterns of activity, dynamic correlations and dynamic high-order correlations to train pattern classifiers to decode (in held-out data) which timepoint in a story participants were listening to.
